# Avelumab, an IgG1 anti-PD-L1 Immune Checkpoint Inhibitor, Triggers NK Cell-Mediated Cytotoxicity and Cytokine Production Against Triple Negative Breast Cancer Cells

**DOI:** 10.3389/fimmu.2018.02140

**Published:** 2018-09-20

**Authors:** Estefanía Paula Juliá, Analía Amante, María Betina Pampena, José Mordoh, Estrella Mariel Levy

**Affiliations:** ^1^Centro de Investigaciones Oncológicas CIO-FUCA, Buenos Aires, Argentina; ^2^Fundación Instituto Leloir, Instituto de Investigaciones Bioquímicas de Buenos Aires (IIBBA)-CONICET, Buenos Aires, Argentina; ^3^Instituto Alexander Fleming, Buenos Aires, Argentina

**Keywords:** Avelumab, triple negative breast cancer (TNBC), PD-L1, ADCC, IL-2, IL-15, IFN-γ

## Abstract

The standard treatment for Triple Negative Breast Cancer (TNBC) patients is cytotoxic chemotherapy, but it is restricted since the duration of response is usually short. Blocking the PD-1/PD-L1 pathway through monoclonal antibodies (mAbs) appears to be a promising therapeutic strategy for TNBC patients. Avelumab is a human IgG1 anti-PD-L1 mAb being tested in clinical trials that may also trigger antibody-dependent cell-mediated cytotoxicity (ADCC) against cancer cells as an additional antitumor activity. In the present work, we studied *in vitro* Avelumab-mediated ADCC against a panel of TNBC cells with different PD-L1 expression using peripheral blood mononuclear cells (PBMC) or purified NK cells from healthy donors. We determined that Avelumab significantly enhanced NK-cell mediated cytotoxicity against TNBC cells and that tumor cells expressing higher levels of PD-L1 were more sensitive to Avelumab-mediated ADCC. IFN-γ treatment upregulated PD-L1 expression in tumor cells but had a variable impact on Avelumab-mediated ADCC, which could be related to the simultaneous effect of IFN-γ on the expression of NK cell ligands. Moreover, IL-2 and IL-15 stimulation of NK cells enhanced Avelumab-triggered cytokine production and degranulation along with increased lytic activity against tumor cells. Improving the treatment of TNBC remains still a considerable challenge. This *in vitro* study suggests that Avelumab-mediated ADCC, independently of the blockade of the PD-1/PD-L1 pathway, could be a valuable mechanism for tumor cell elimination in TNBC. Avelumab combination with immunomodulators such as IL-15 or IL-2 could be taken into consideration to increase the therapeutic efficacy of Avelumab in TNBC.

## Introduction

Triple-negative breast cancer (TNBC) is defined by the lack of expression of both estrogen and progesterone receptors and the absence of ERBB2 (HER2) over-expression. TNBC is a heterogeneous disease that accounts for 10–15% of all breast cancers (BC) (1) and is an aggressive subtype associated with poor outcomes compared to other subtypes (2, 3). The standard treatment for TNBC patients is cytotoxic chemotherapy, but it is restricted since the duration of response is usually short and rapid relapses are very common (3, 4).

TNBC has been divided into six molecular subtypes; one of them is the immunomodulatory subtype characterized by an enriched expression of genes involved in immune cell processes (5), a high immune cell infiltration in the tumor stroma and a high mutation rate (6, 7). Additionally, several studies have confirmed the predictive value of tumor-infiltrating lymphocytes (TILs) in neoadjuvant chemotherapy for TNBC (8–10). These features make TNBC patients suitable candidates for immunotherapy (11, 12).

The programmed cell death protein 1 (PD-1) is a negative regulator that limits the activity of T cells at a variety of stages of the immune response, particularly in the effector phase within tissues and tumors (13). PD-1 inhibits effector T-cell activity when it interacts with its two ligands: PD-L1 and PD-L2 (14). PD pathway-mediated evasion of tumor immunity is described as “adaptive resistance” since PD-L1 is absent in most normal tissues, while IFN-γ can induce its expression in virtually any nucleated cells (13–17). In some cancers, PD-L1 expression is driven constitutively by aberrant signaling pathways or chromosomal alterations (18). For instance, PTEN mutations generating PI3K–AKT pathway activation in some glioblastoma cases and 9p24.1 gene translocations or amplifications in certain lymphomas can result in extensive expression of PD-L1 and PD-L2 on the surface of the majority of tumor cells (18). The PTEN/PI3K pathway is relevant in BC because PTEN loss and PIK3CA mutations have been found in around 30 and 40% of primary breast tumors, respectively (19). Mittendorf et al. have shown that PTEN knockdown led to an increased cell membrane PD-L1 expression and PD-L1 transcripts in BC cell lines, suggesting transcriptional regulation (20). Recently, PD-L1 expression in primary BC tumors has been detected mainly in TNBC subtype with positive correlation with the amount of TILs and the presence of peritumoral tertiary lymphoid structures (TLS) (21). In this regard, PD-1/PD-L1 pathway blockade appears to be a promising therapeutic strategy for this tumor type.

Avelumab is a human IgG1 monoclonal antibody directed to PD-L1 that blocks the binding between PD-1 and PD-L1 without affecting PD-1/PD-L2 interactions. Avelumab was tested in a phase 1 trial (JAVELIN Solid Tumor; NCT01772004) in patients with metastatic BC refractory to or progressing after standard-of-care therapy (22). Avelumab had an acceptable safety profile and clinical activity in a subset of metastatic BC patients. Patients with PD-L1 expression in tumor-associated immune cells may present higher probability of clinical response to Avelumab (22). The data obtained in this and other studies suggest that it is possible to achieve a long-lasting clinical response with anti-PD-1/PD-L1 as monotherapy in a subgroup of metastatic BC patients, mainly TNBC (23, 24).

Moreover, unlike other PD-L1 blocking mAbs, Avelumab can potentially mediate Ab-dependent cell cytotoxicity (ADCC) against tumor cells (25). ADCC is defined as the immune mechanism through which Fc-receptor-bearing effector cells can kill target cells that have surface antigens complexed with an antibody. Three types of Fc receptors are involved in mediating IgG-dependent ADCC: FcγRI (CD64), FCγRII (CD32), and FcγRIIIA (CD16). Of these, FcγRIIIA (CD16) is often invoked as the main receptor involved, as it is expressed predominantly by Natural Killer (NK) cells (26).

NK cells are lymphocytes of the innate immunity that contribute with early responses against virus-infected and transformed cells (27). NK cell activation is controlled by a repertoire of germ-line encoded surface receptors that interact with their ligands on the target cell surface (28). The integration of signals from adhesion molecules and activating receptors induces the secretion of cytotoxic mediators (i.e., granzyme B and perforin) and the production of pro-inflammatory cytokines (e.g., IFN-γ and TNF-α) and diverse chemokines (29). Mature NK cells express at least one inhibitory receptor specific for self-MHC class I molecules (MHC-I) as a protective mechanism, repressing NK-cell activation against non-transformed cells (27, 30). Cytokines enhancing NK-cell proliferation and function such as IL-2 and IL-15 can be used to potentiate NK cell-mediated ADCC against antibody-coated tumor cells (31), offering the potential for multiple combinatorial immunotherapy strategies against cancer.

Recently, Avelumab-dependent cell cytotoxicity was demonstrated in an *in vitro* setting against several tumor models (25). However, there is still no clinical evidence available to show the contribution of ADCC to the clinical activity of Avelumab. Moreover, it has been shown that PD-L1 is also expressed by immune cells. However, a phase I trial with 28 patients showed the lack of any significant effect on the peripheral blood frequency of several immune cell subsets, even those expressing PD-L1, following multiple cycles of Avelumab. In addition, *ex vivo* experiments showed that NK cells isolated from metastatic NSCLC patients mediated ADCC triggered by Avelumab against human lung tumor cell lines but not against autologous PBMC, even when sorted to enrich for PD-L1 expression (32).

Due to the few possibilities of treatment in TNBC, in the present work we evaluated *in vitro* Avelumab-mediated ADCC against TNBC cell lines with different basal or IFN-γ-induced expression of PD-L1. We also investigated the effect of IL-2 and IL-15 on NK cell activation and cytokine production triggered by Avelumab.

## Methods

### Cell lines and cell culture

IIB-BR-G cell line has been established from a primary infiltrating ductal carcinoma (33). MDA-MB-231 (ATCC® HTB-26™), MDA-MB-468 (ATCC® HTB-132™), BT-549 (ATCC® HTB-122™) and Hs578T (ATCC® HTB-126™) were acquired from ATCC. All cell lines were grown at 37°C in a humid atmosphere containing 5% CO_2_ with Dulbecco's Modified Eagle Medium: Nutrient Mixture F-12 (DMEM/F12, Thermo-Fisher) except for BT-549 that was grown with RPMI-1640 Medium (Thermo-Fisher). Culture media were supplemented with 10% fetal calf serum (FCS), 2 mM L-glutamine and 10 μg/ml insulin. When indicated, cells were treated at 60–80% confluence with 10 IU/ml of recombinant human IFN-γ (Imukin-Boehringer Ingelheim) for 24 h and then harvested using EDTA/PBS.

### Immunofluorescence analysis by FACS

Direct immunofluorescence staining was performed on TNBC cells for 30 min at 4°C using the following mAbs: FITC anti-MHC class I (clone G46-2.6), PE anti-CD112 (clone R2.5025) and PE anti-MICA/B (clone 6D4) from BD Biosciences; PE anti-CD155 (clone SKII.4) and APC anti-PDL1 (clone 29E.2A3) from BioLegend; PE anti-HLA-G (clone MEM-G/9) from Abcam; and their isotype-matched control mAbs. Indirect immunofluorescence was performed using anti-HLA-E (clone MEM-E/08, Abcam) or mouse monoclonal IgG1. Primary antibodies were incubated for 1 h at 4°C. After washing, cells were incubated for 1 h at 4°C with the secondary PE-labeled mAb. For dead cell exclusion, cells were stained with 7-Aminoactinomycin D (7-AAD) for 20 min on ice. Cells were acquired in a FACSCanto II flow cytometer (BD), and data were analyzed using FlowJo software (Tree Star). Results were expressed as a percentage of positive cells or normalized Median fluorescence intensity (MFI): MFI of cells stained with specific mAb/MFI of cells stained with isotype control. Fold change in expression after IFN-γ exposure was calculated as: normalized MFI of IFN-γ treated cells/normalized MFI of untreated cells.

### Isolation of human cells and stimulation

Peripheral blood mononuclear cells (PBMC) from healthy donors were obtained by Ficoll–Paque PLUS (GE Healthcare) density gradient centrifugation and cryopreserved in FCS plus 10% dimethyl sulfoxide (DMSO). All donors signed an informed consent approved by the Institutional Review Board of the Instituto Alexander Fleming. PBMC effectors were thawed the evening before the assay and allowed to rest overnight (ON) in RPMI-1640 medium containing 10% FCS. When indicated, 1,000 IU/ml IL-2 or 10 ng/ml IL-15 (PreproTech) was added during the ON incubation and then washed out before the assay. For some experiments, NK cells were isolated from PBMC using NK cell Isolation Kit (Miltenyi Biotec) following the manufacturer's instructions and allowed to rest ON.

### Lysis and ADCC assay

TNBC cells used as targets were labeled with 10 μM of Calcein-acetoxymethyl (Calcein-AM; Molecular Probes, Invitrogen) for 30 min at 37°C, washed twice and resuspended in serum-free assay medium (AIM V, Thermo Fisher). Five thousand target cells/well were seeded in 96-well plates. Effector cells were purified NK cells or PBMC normalized by percentage of NK cells. The cytotoxicity assays were performed at different NK cell:breast cancer cell (NK:BC) ratios in triplicate, with 1 μg/ml of Avelumab (Merck Serono) or human myeloma IgG1 isotype control mAb (catalog I 5154, Sigma-Aldrich). For some experiments, a humanized IgG1 anti-PD-L1 mAb engineered to avoid binding to human Fcγ receptors through a N298A mutation (Atezolizumab, Roche) (34) was used to assess the effect of PD-L1 blockade on NK cell cytotoxicity independent of ADCC. For spontaneous and maximum release, targets were incubated without effectors in assay medium alone or assay medium plus 1% Triton X-100, respectively. After incubation for 4 h at 37°C in 5% CO_2_, plates were centrifuged, and Calcein release was measured in supernatants by fluorimetry (OPTIMA, BMG Labtech). The percentage of specific lysis was calculated as (experimental release – spontaneous release)/(maximum release – spontaneous release) ^*^ 100.

### CD107a degranulation and cytokine production assay

2.5 × 10^5^ PBMC were cultured with TNBC cells in the presence of 1 μg/ml of Avelumab or human IgG1 isotype control mAb in 96-well plates with AIM V medium. The number of target cells was calculated according to the percentage of NK cells, so the co-cultures were performed at 1:1 NK cell:breast cancer cell (NK:BC) ratio. Cells were incubated for 6 h at 37°C in 5% CO_2_, with the addition of FITC anti-CD107a (clone H4A3, BD) from the beginning of the assay and Protein Transport Inhibitor (Golgi Stop, BD) after the first hour. Cells were harvested, washed and labeled with BV421 anti-CD56 and APC-H7 anti-CD3 (clones NCAM16.2 and SK7, BD) for 15 min at room temperature. After that, cells were fixed and permeabilized using Fixation and Permeabilization kit (BD) according to manufacturer's protocol, and then labeled with APC anti-IFN-γ and PE anti-TNF-α (clones 4S.B3 and MAb11, BD) for 30 min at 4°C. Cells were acquired in a FACSCanto II flow cytometer, and data were analyzed using FlowJo software. A representative gating strategy is shown in Supplementary Figure 2. The results were expressed as the percentage of IFN-γ+, TNF-α+, or CD107a+ cells after gating on total NK cells or CD56^dim^ and CD56^bright^ NK cell subsets. Basal degranulation and IFN-γ/TNF-α production were determined in the absence of target cells and are shown in the corresponding graphs.

### Statistical analysis

GraphPad Prism 7.0 was used for graphs and paired t-test analysis. The comparison of multiple treatments was done with Infostat 2017 software (35) ANOVA with randomized block design was used to analyze data, considering treatments with mAbs (IgG1 or Avelumab), cytokines (IL-2 or IL-15) and their interaction as fixed factors and donors as a random factor (blocks); α = 0.05. Homoscedasticity and normality of residuals were analyzed by visual assessment of plots. If homoscedasticity was not achieved, models were fitted by the inclusion of the VarIdent, VarExp, or VarPower variance structure to the random part of the model (36). The best variance structure was chosen by comparison of Akaike's and Bayesian's Information Criteria. A p < 0.05 was considered to be statistically significant. EulerAPE was used for drawing Area-proportional Venn diagrams (37).

## Results

### Avelumab triggered NK cell-mediated cytotoxicity against TNBC cells depending on PD-L1 expression by tumor cells

**Figure 1:**
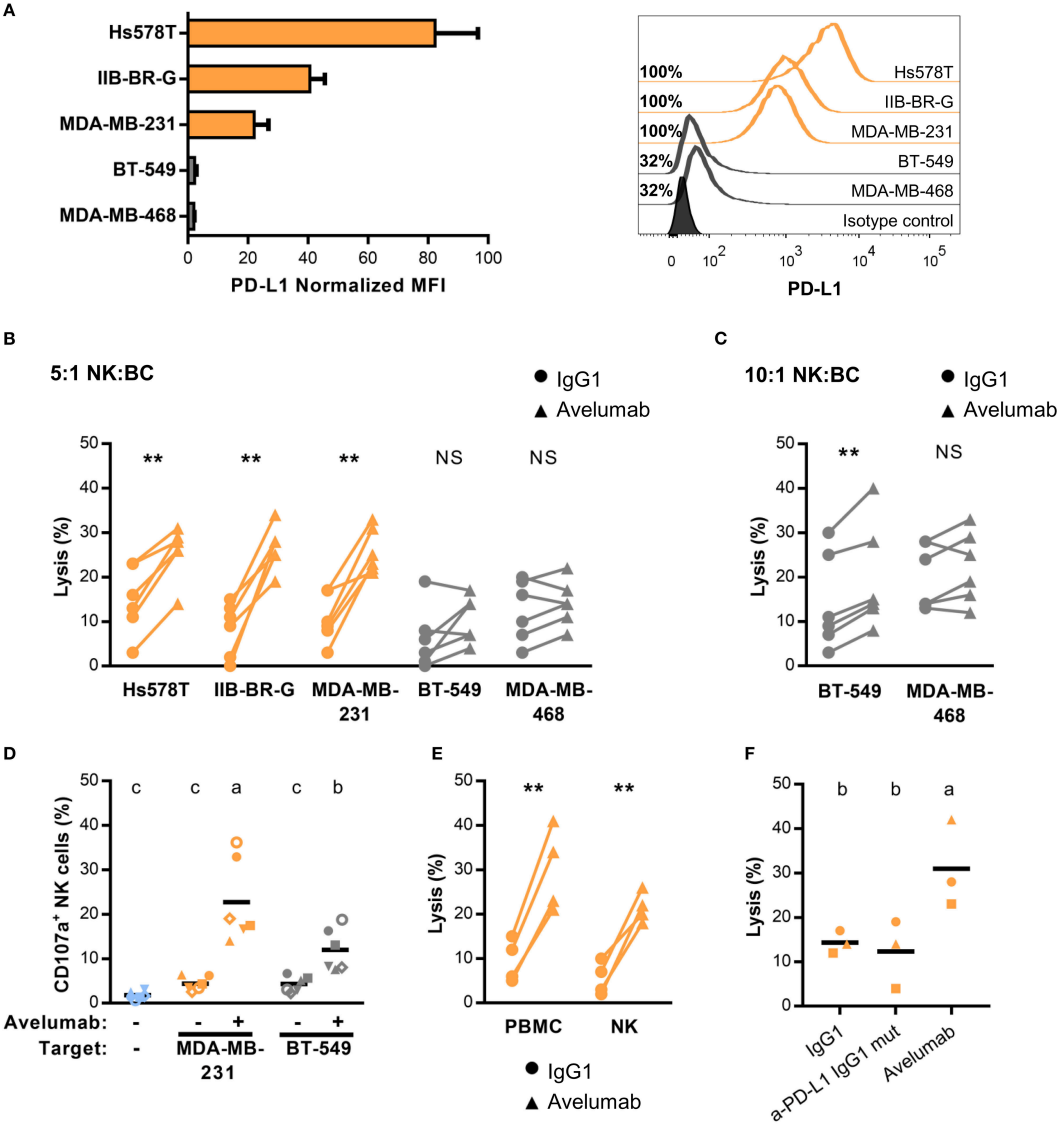
PD-L1 expression and Avelumab mediated-ADCC of TNBC cell lines **(A)** PD-L1 expression in five TNBC cell lines represented as normalized MFI (MFI of cells stained with specific mAb/ MFI of cells stained with isotype control) in the left, and percentage of positive cells with representative histograms in the right. Cells were analyzed by FACS after staining with APC anti-PDL1 (clone 29E.2A3). In all of the figures, cells with higher expression of PD-L1 are shown in orange and the ones with lower expression, in gray. **(B)** Percentage of lysis against the five TNBC cell lines coated with 1 μg/ml of isotype control (IgG1) or Avelumab, at 5:1 NK:BC ratio. **(C)** Percentage of lysis against BT-549 and MDA-MB-468 cells (low PD-L1 expression) coated with IgG1 or Avelumab, at 10:1 NK:BC ratio. In **(B,C)** PBMC from the same six donors were used as effector cells for all the cell lines; ^**^p < 0.01 (paired t-test). **(D)** Percentage of degranulating NK cells (CD3^−^CD56^+^CD107a^+^) against MDA-MB-231 (high PD-L1 expression) and BT-549 (low PD-L1 expression) coated with IgG1 or Avelumab using PBMC from the same six donors (each symbol represents an individual donor). Basal degranulation without target cells is shown in light-blue symbols. Bars with different letters are statistically different,p < 0.05 (ANOVA). **(E)** Percentage of lysis against MDA-MB-231 cells coated with IgG1 or Avelumab using PBMC or purified NK cells as effector cells (n = 4); ^**^p < 0.01 (paired t-test). **(F)** Percentage of lysis against MDA-MB-231 cells coated with IgG1, a mutant IgG1 anti-PD-L1 mAb that does not bind to human Fcγ receptors (Atezolizumab), or Avelumab using PBMC as effectors at 5:1 NK:BC ratio (n = 3). Bars with different letters are statistically different, p < 0.05 (ANOVA).

PD-L1 in BC has been reported to be expressed mostly in hormone receptor-negative tumors (20). We tested cell surface PD-L1 expression in five TNBC cell lines by flow cytometry, four of them previously characterized (20). We found variable levels of PD-L1 expression; cell lines Hs578T, IIB-BR-G, and MDA-MB-231 expressed high levels of PD-L1, with 100% of cells positive for PD-L1 and a normalized MFI over 20. The other two cell lines (BT-549 and MDA-MB-468) expressed lower PD-L1 levels, with ~30% of cells positive for PD-L1 and a low normalized MFI (Figure 1A).

Avelumab is an IgG1 mAb directed to PD-L1 that, besides its function in immune checkpoint blockade, can mediate *in vitro* ADCC against tumor cells that express its target, with NK cells being the main effector cell population (25). We studied Avelumab ability to trigger ADCC against these five TNBC cell lines with different levels of PD-L1 expression. Using PBMC as the source of effector cells, Avelumab significantly increased tumor cell lysis compared to isotype control in the three cell lines with high PD-L1 expression at NK:BC ratio of 5:1 (Figure 1B). Regarding the cell lines expressing lower PD-L1 levels, BT-549 was killed by ADCC only at a higher NK:BC ratio of 10:1, while Avelumab did not increase MDA-MB-468 cell lysis in either of the two conditions (Figure 1C). In concordance with these results, Avelumab opsonization of TNBC cells increased NK cell degranulation (measured as CD107a cell surface expression) compared to isotype control. CD107a induction by Avelumab was significantly higher against MDA-MB-231 (PD-L1 high) than against BT-549 (PD-L1 low) (Figure 1D). We also evaluated cytotoxicity against MDA-MB-231 cells using NK cells purified from PBMC which corroborated that NK cells were major effectors of Avelumab-mediated ADCC (Figure 1E). Blocking PD-L1 with an IgG1 mAb engineered to avoid binding to human Fcγ receptors did not have a significant effect on cytotoxicity against MDA-MB-231 cells compared to isotype control (Figure 1F).

### Modulation of PD-L1 and NK cell ligand expression in TNBC cell lines after treatment with IFN-γ had a variable impact on avelumab-mediated ADCC

**Figure 2:**
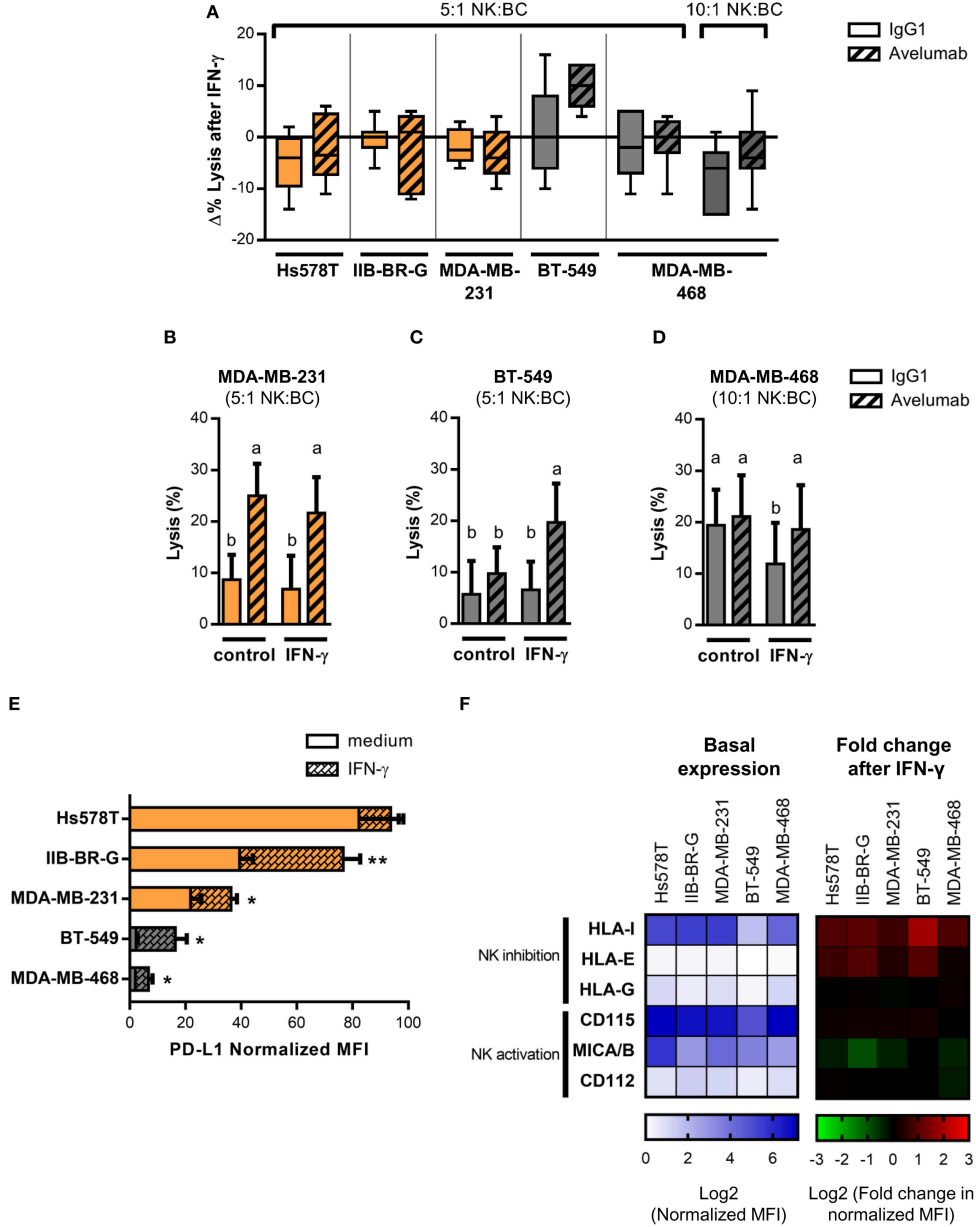
IFN-γ effect on PD-L1 and NK cell ligand expression and susceptibility to Avelumab-mediated ADCC of TNBC cell lines. TNBC cells lines were treated with 10 U/ml IFN-γ for 24 h or left untreated before being used in the assays. In all of the figures, cells with higher basal expression of PD-L1 are shown in orange and the ones with lower basal expression, in gray. Lysis of IFN-γ treated and untreated cancer cells coated with IgG1 or Avelumab was evaluated using PBMC as effector cells at 5:1 or 10:1 NK:BC ratios (n = 6–7): **(A)** Changes in lysis (Δ % Lysis) of treated compared to untreated cancer cells were quantified for each donor and results are shown as box plots; **(B)** Percentage of lysis against MDA-MB-231 cells at 5:1 NK:BC ratio; **(C)** Percentage of lysis against BT-549 cells at 5:1 NK:BC ratio; **(D)** Percentage of lysis against MDA-MB-468 cells at 10:1 NK:BC ratio. Bars with different letter are statistically different, p < 0.05 (ANOVA). **(E)** PD-L1 expression in IFN-γ treated and untreated TNBC cell lines represented as normalized MFI (MFI of cells stained with specific mAb/MFI of cells stained with isotype control). ^*^p < 0.05; ^**^p < 0.01 (paired t-test). **(F)** Heat-map representing basal expression of NK cell ligands in TNBC cell lines, showed as log_2_ normalized MFI (left) and heat-map representing fold change in NK cell ligand expression after IFN-γ treatment of TNBC cell lines, showed as log_2_ fold change in normalized MFI (right).

IFN-γ has been reported to induce changes in the expression of several tumor cell ligands for NK cell activating and inhibitory receptors with different results on cancer cell sensitivity to NK cell-mediated lysis (38, 39). Moreover, PD-L1 expression has been described to increase in response to both type I and II interferons produced during an active antitumor immune response and could provide a mechanism of tumor escape from T cell responses (17). Therefore, we studied the effect of IFN-γ pre-treatment of TNBC cells on NK cell cytotoxicity. Changes in lysis and ADCC (Δ% Lysis) of treated compared to untreated cancer cells were quantified for each donor (Figure 2A). While exposure of high PD-L1 expressing cell lines to IFN-γ had no significant impact on Avelumab- triggered ADCC (Figures 2A,B), pre-treatment of BT-549 cells (low PD-L1 level) with IFN-γ significantly increased ADCC levels (Figures 2A,C). In concert, NK cell degranulation against BT-549 cells coated with Avelumab was higher when target cells had been exposed to IFN-γ (Supplementary Figure 1).

On the other hand, MDA-MB-468 (low PD-L1 level) cells were not sensitive to ADCC at 5:1 NK:BC ratio even when treated with IFN-γ (Figure 2A), so we tested a higher NK:BC ratio of 10:1. IFN-γ significantly reduced lysis against isotype control-coated cells, but Avelumab recovered cytotoxicity to the level of non-treated cells (Figures 2A,D).

We searched for changes in the phenotype of TNBC cells that could explain these effects of IFN-γ on NK cell lysis, starting with PD-L1 expression. IFN-γ induced PD-L1 in all cell lines except for Hs578T that had already the highest basal level of PD-L1 (Figure 2E). BT-549 showed the greatest change in PD-L1 expression (a six-fold increase) which could account for the increased sensitivity to Avelumab-triggered ADCC. Next, we studied changes in the expression of NK cell inhibitory (MHC-I, HLA-E, and HLA-G) and activating (MICA/B, CD112, and CD155) ligands. The most upregulated NK inhibitory ligand was MHC-I, which increased at least 1.5-times in all cell lines, followed by HLA-E. On the other hand, NK activating ligands were either not modulated or tended to be downregulated (Figure 2F).

In summary, Avelumab-mediated ADCC was increased or maintained when tumor cells were exposed to IFN-γ. The balance between the changes in PD-L1 levels and NK cell ligands could explain the different results observed in the five cell lines.

### IL-2 and IL-15 stimulation of NK cells enhanced avelumab-triggered cytokine production and degranulation

**Figure 3:**
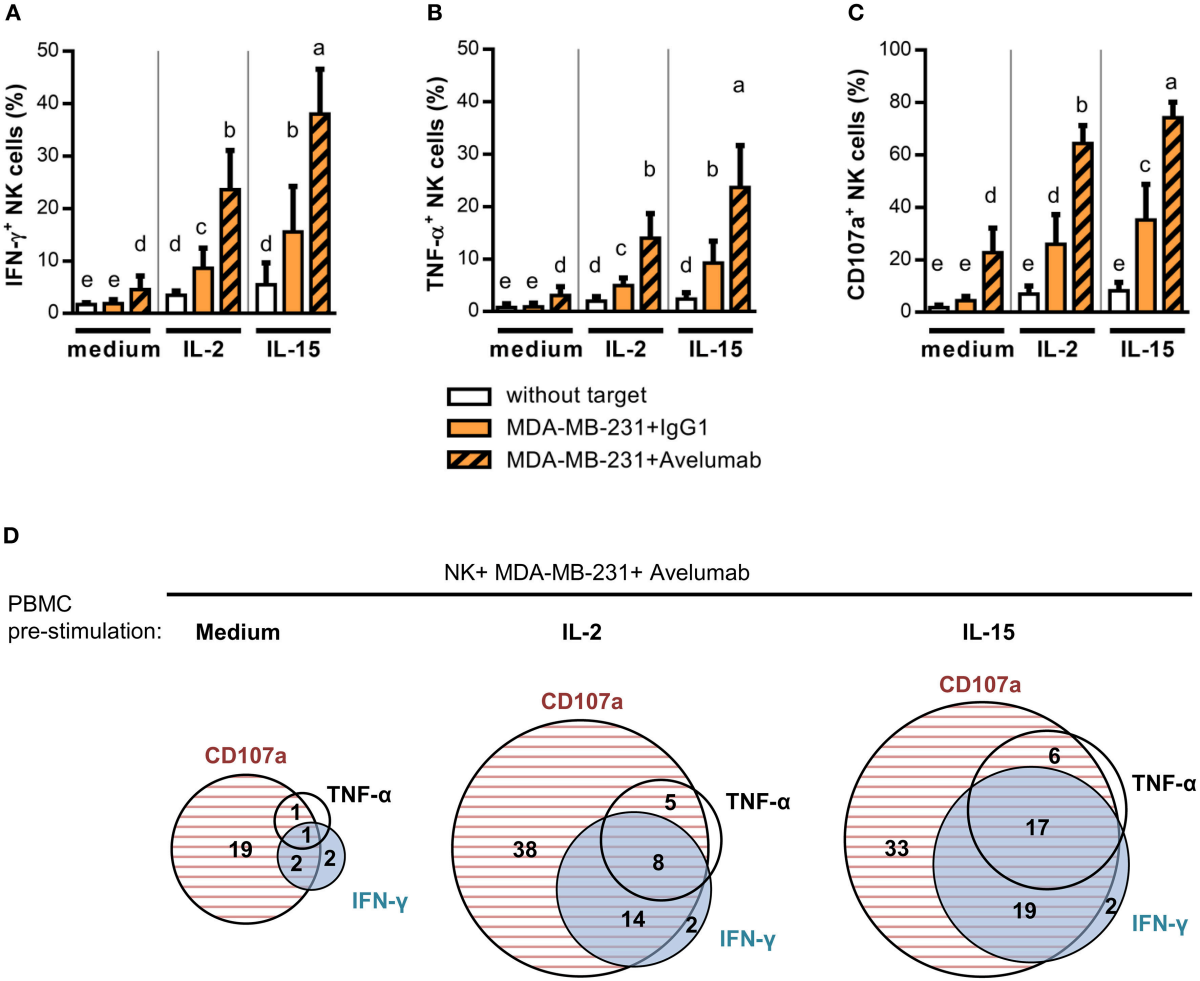
IL-2 and IL-15 effect on NK cell degranulation and cytokine production triggered by Avelumab coated TNBC cells. PBMC were stimulated ON with 1,000 U/ml IL-2 or 10 ng/ml IL-15, or left untreated, and then cultured without target cells or with MDA-MB-231 coated with IgG1 or Avelumab to study degranulation and cytokine production as described in Materials and Methods: **(A)** Percentage of NK cells that produced IFN-γ (CD3^−^CD56^+^IFN-γ^+^); **(B)** Percentage of NK cells that produced TNF-α (CD3^−^CD56^+^TNF-α^+^); **(C)** Percentage of NK cells that degranulated (CD3^−^CD56^+^CD107a^+^). Bars with different letter are statistically different, p < 0.05 (n = 6, ANOVA). **(D)** Area-Proportional Venn diagrams were used to represent the percentage of NK cells expressing IFN-γ, TNF-α, CD107a or co-expressing these markers against MDA-MB-231 cells coated with Avelumab. Effector cells were rested (left) or stimulated ON with IL-2 (middle) or IL-15 (left) before the assay.

It has long been observed that NK cell activation through FcγRIIIa promotes pro-inflammatory cytokine secretion. This activity known as Ab-dependent cytokine release—ADCR—has been postulated to influence other immune cells in proximity and to be concomitant to ADCC (40). Here, we studied pro-inflammatory cytokines produced as a consequence of Avelumab-mediated NK cell activation. Avelumab slightly induced IFN-γ and TNF-α production by NK cells against MDA-MB-231 cells compared to isotype control (Left panel of Figures 3A,B, respectively).

With the aim of studying immune modulators to augment Avelumab antitumor activities, we assessed the effect of treating effector cells with IL-2 or IL-15. Both treatments increased IFN-γ (Figure 3A) and TNF-α (Figure 3B) production and NK cell degranulation (Figure 3C) against target cells coated with isotype control compared to effector cells alone, and all of these activities were greatly augmented by Avelumab. Of note, IL-15 stimulated NK cell degranulation and cytokine production more than IL-2. As shown in Figure 3D, NK cells that produced IFN-γ and/or TNF-α were mainly the ones that had degranulated.

We next analyzed pro-inflammatory cytokine production within CD56^dim^ and CD56^bright^ NK subsets separately. In the absence of the pre-stimulation of effector cells, the low level of pro-inflammatory cytokines induced by Avelumab was mainly produced by CD56^dim^ NK cells. Meanwhile, when effector cells were stimulated with IL-2 or IL-15 in combination with Avelumab-coated cells, both CD56^bright^ and CD56^dim^ subsets produced cytokines (Supplementary Figure 2).

### Stimulation with IL-2 and IL-15 increased cytotoxicity of TNBC cells

**Figure 4:**
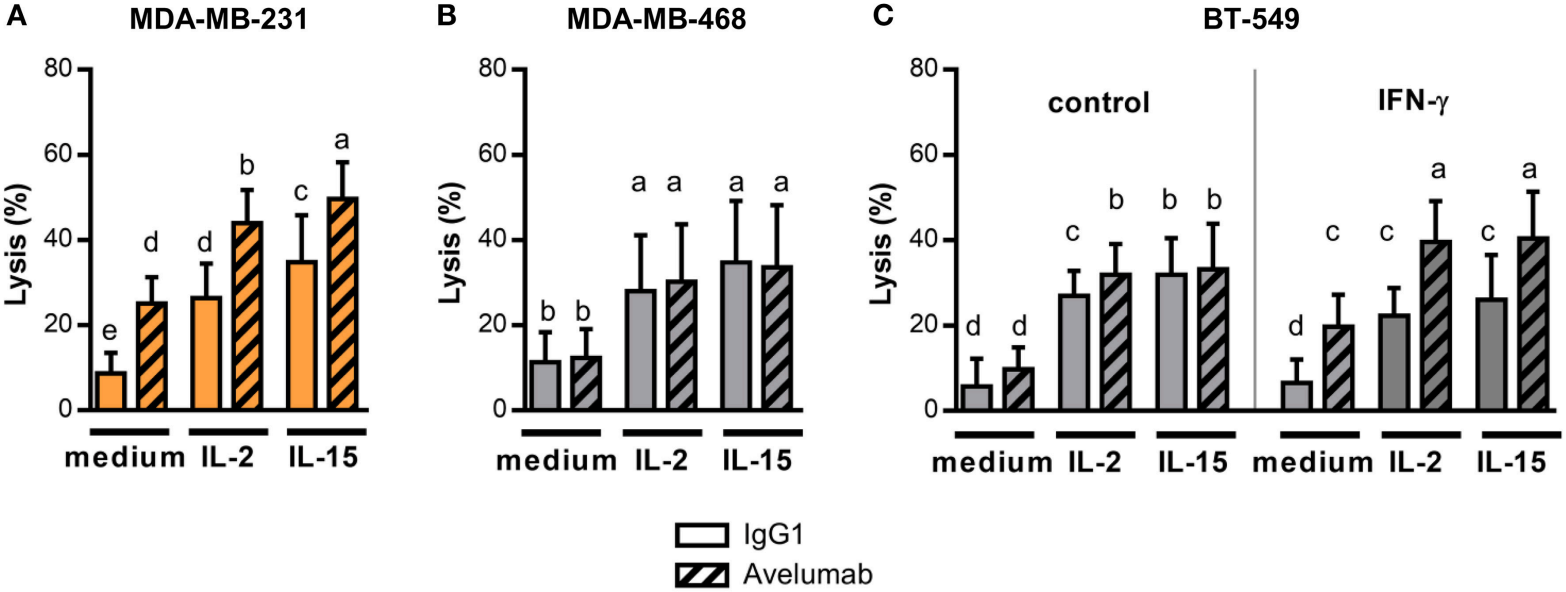
IL-2 and IL-15 effect on Avelumab-mediated ADCC of TNBC cell lines. Cytotoxic assays were performed at 5:1 NK:BC ratio with PBMC un-stimulated or pre-stimulated ON with 1,000 U/ml IL-2 or 10 ng/ml IL-15: **(A)** Percentage of lysis against MDA-MB-231 cells (high basal PD-L1 expression) coated with IgG1 or Avelumab; **(B)** Percentage of lysis against MDA-MB-468 cells (low basal PD-L1 expression) coated with IgG1 or Avelumab; **(C)** Percentage of lysis against BT-549 cells (low basal PD-L1 expression) coated with IgG1 or Avelumab, un-treated (left) or pre-treated with IFN-γ (right). Bars with different letters are statistically different, p < 0.05 (n = 6, ANOVA).

Next, we studied IL-2 or IL-15 effect on lysis of TNBC cells, starting with high PD-L1 expressing cell lines. IL-2 or IL-15 addition to effector cells increased Avelumab-mediated ADCC against MDA-MB-231 cells compared to untreated effectors (Figure 4A). Noteworthy, the effect of IL-2/IL-15 and Avelumab combination was additive, as the same magnitude of increase in lysis by IL-2/IL-15 was obtained when target cells were coated with Avelumab or with isotype control (test for interaction in ANOVA with two fixed factor, cytokine and mAb: *p* = 0.67).

Then, we analyzed IL-2 or IL-15 effect on cytotoxicity against the low PD-L1 expressing cell lines that were slightly or not sensitive to ADCC in basal conditions (Figures 1B,C). Both cytokines increased lysis of target cells to a similar extent when coated with Avelumab or with isotype control (Figure 4B for MDA-MB-468 and Figure 4C left panel for BT-549). So, IL-2 or IL-15 increased the cytotoxic activity of NK cells but did not to increase the sensitivity of tumor cells to Avelumab-mediated ADCC.

Taking into account that Avelumab treatment in combination with IL-2 or IL-15 may result in tumor cells being exposed to higher levels of IFN-γ derived from NK cells or CD8 T cells (41); we further studied *in vitro* the effect of combining both treatments. We focused on BT-549 cell line which displayed the greatest change in PD-L1 expression after IFN-γ exposure (Figure 2E). As we previously shown, IFN-γ augmented sensitivity of BT-549 to Avelumab-mediated ADCC, and IL-2 or IL-15 pre-treatment significantly increased ADCC compared to untreated effectors (Figure 4C right panel).

## Discussion

Chemotherapy is the current standard treatment for TNBC. Even though TNBCs are highly sensitive to chemotherapy, patients have high rates of relapse, which has been described as the “triple-negative paradox” (2, 42, 43). Therapeutically targeting TNBC with mAbs that block the PD-1/PD-L1 axis is supported by diverse novel studies. Mittendorf et al. (20) described higher expression of PD-L1 in TNBC than in hormone receptor-positive BC; the analysis on tissue microarrays showed around 20% of primary TNBC tumors expressing PD-L1. Moreover, several studies have reported that TNBCs present higher level of TILs compared with hormone receptor-positive BC (9), with prognostic significance in TNBC (44). In addition, TNBC present genomic instability and high rates of genetic mutations, which can implicate the generation of more neoantigens and augmented immunogenicity (45, 46). Nowadays, several mAbs are being studied to block PD-1/PD-L1 axis in TNBC; preliminary data from clinical trials presented promising results for patients with advanced stage/metastatic TNBC. Patients who responded to the treatment had a favorable prognosis and often showed a significant increase in the overall survival. Still, the objective response rate was relatively low. Therefore, it is necessary to find strategies to enhance the response to this therapy in TNBC and to transform the non-responders into responders (47).

Unlike other anti-PD-L1 mAbs, Avelumab was designed as IgG1 to trigger ADCC against tumor cells (25). ADCC involves ligation of CD16 expressed by NK cells with the Fc region of mAbs. Tumor antigen-targeting mAbs have demonstrated success in the treatment of various malignancies and are now widely used in the clinic (48). With the aim to characterize the Fcγ-mediated mechanism of action played by Avelumab in TNBC, we studied *in vitro* Avelumab-dependent cellular cytotoxicity and cytokine release against five cell lines that present a spectrum of PD-L1 expression levels. We demonstrated that Avelumab triggered ADCC against TNBC cells expressing certain levels of PD-L1 with a significant increase in tumor cell lysis compared to isotype control when PBMC or purified NK cells were used as effector cells. We did not perform a correlation analysis between PD-L1 expression and ADCC sensitivity because of the limited number of cell lines but, in this regard, Boyerinas developed a PD-L1 score that integrates % PD-L1–positive cells and MFI, using the same clone of anti-PD-L1 mAb for staining (25). We calculated this score for the cell lines used in the present work. As they described, scores ≥ 6 (Hs578T and IIB-BR-G) were predictive of ADCC sensitivity, whereas scores ≤ 4 (BT-549 and MDA-MB-468) were predictive of low or non-ADCC sensitivity. To take into account, the optimal isotype control would have been an IgG1 produced as Avelumab, with an almost identical Fc-amino acid sequence, since the isotype control used could have had different binding to FcγRIIIa than Avelumab due to differences in fucosylation.

In the experimental conditions of this study, we detected very low or non-PD-1 expression on NK cells of healthy donors (data not shown) and blocking PD-L1 with an IgG1 mAb engineered to avoid binding to human Fcγ receptors did not increase cytotoxicity. However, recent studies have shown that PD-1 is upregulated on NK cells of patients with various tumors (49–52) functioning as an inhibitory regulator of NK cells. In that context, blockade of PD-1/PD-L1 interaction increased NK cell activity against tumor cells expressing PD-L1. This could be seen as another path for the action of Avelumab, analogous to the mode of action on T lymphocytes.

One factor that can be involved in the inter-individual variability in NK cell-mediated ADCC is the CD16 polymorphism. Although this association has been determined in studies involving different tumor antigen-targeting mAbs of IgG1 isotype (Rituximab, Trastuzumab, and Cetuximab), it is still controversial in different settings (53). Preliminary *in vitro* results have shown higher Avelumab-mediated ADCC with NK cells from volunteers with the V/V genotype (25). Clinical studies are needed to investigate if there is an association between FcγRIIIa polymorphism and clinical response in patients treated with Avelumab.

It has been described in solid tumors that cytotoxic TILs upregulate PD-1 expression and secrete IFN-γ when they encounter tumor antigens, which triggers an adaptive response to IFN-γ with a consequent upregulation of PD-L1 expression in tumor cells and infiltrating immune cells in the vicinity (54, 55). We studied the effect of IFN-γ treatment on TNBC cells, determining that it significantly augmented PD-L1 expression in 4 out of 5 cell lines, but at the same time it modulated the expression of some NK cell ligands, with MHC-I being the most upregulated. Besides, it is possible that IFN-γ modulated NK cell ligands other than the ones analyzed in this study which could have differentially impacted on NK cell cytotoxicity. As was observed by Boyerinas, IFN-γ did not always enhance ADCC, which could probably be explained by the balance between the expression of PD-L1 and NK cell ligands after IFN-γ treatment (25). Only in the case of BT-549 cells, IFN-γ significantly increased sensitivity to Avelumab triggered ADCC, which could be explained by a six-fold increase in PD-L1 expression. Recently, Aquino-López (38) studied a panel of 22 pediatric-tumor cell lines and showed that IFN-γ led to an increased resistance to NK cells for six of them, while it resulted in augmented sensitivity for three of them. Several NK cell ligands were upregulated, but the results of that study suggested that the effect of IFN-γ on NK cell-mediated lysis of tumor cells was mainly associated with changes in MHC-I and ICAM-1 expression. In our study, lysis against TNBC cells in the absence of Avelumab was rather low at the NK:BC ratios used and tend to diminish with IFN-γ treatment for MDA-MB-468 cell line. This negative effect of IFN-γ was more marked when effector cells were stimulated with IL-2/IL-15 and was observed for other cell lines in addition to MDA-MB-468 (data not shown). However, Avelumab mediated cytotoxicity was maintained or even increased under these unfavorable conditions for NK cell lysis.

It has long been observed that NK cell activation through FcγRIIIa promotes IFN-γ and TNF-α secretion (40). Regarding tumor antigen-targeting mAbs, it was demonstrated that Trastuzumab-coated cancer cells could induce IFN-γ production by human NK cells, especially in the presence of IL-12, and IFN-γ release was considered an important factor for Trastuzumab-antitumor activity (56). Additionally, it was shown that mAb-mediated promotion of IFN-γ release induced dendritic cell maturation and higher antigen presentation, which is supposed to augment cross-presentation to CD8 T cells, thus connecting the innate and adaptive immune responses (57). Here, we demonstrated for the first time that Avelumab was able to trigger IFN-γ and TNF-α production by NK cells against TNBC cells *in vitro*. Both activities were concomitant with Avelumab-mediated NK cell degranulation and occurred mainly in the CD56^dim^ subset.

Moreover, looking for strategies to improve antitumor activities mediated by NK cells, we previously determined that IFN-γ release was promoted by IL-2/IL-15 in an *in vitro* model testing Cetuximab-mediated ADCC against TNBC cells (58). In the present work, both IL-2 and IL-15 promoted a significant augmentation of IFN-γ and TNF-α production by both CD56^dim^ and CD56^bright^ NK cell subsets when TNBC cells were coated with Avelumab. IL-2/IL-15 stimulation also increased lysis and ADCC against TNBC cells but did not increase the sensitivity of low PD-L1 expressing cells to Avelumab-mediated ADCC. Interleukins promote the activation, differentiation, proliferation, and/or survival of lymphocytes. While IL-2 stimulates almost all T cells, including Treg cells, and most NK cells (59), IL-15 activates mainly CD8+ T cells and non-terminally differentiated NK cells (60, 61). Lately, many approaches have been described to improve the *in vivo* half-life and efficacy of IL-15, principally by producing IL-15/IL-15Rα conjugates called IL-15 superagonist (62). This renovates the prospect of using IL-15 as a cancer immunotherapeutic drug and combining it with other immuno-oncological agents. Pre-clinical models have demonstrated enhanced anti-tumor responses by combining IL-15 or IL-15/IL-15Rα with checkpoint blocking antibodies targeting murine PD-L1 (62). Recently, a phase I clinical trial has shown that administration of IL-15 superagonist ALT-803 in combination with nivolumab is safe and tolerable (63). Regarding other immunomodulators, pre-clinical (64, 65) and clinical (NCT02994953) studies are investigating NHS-muIL12 and Avelumab combination therapy in different tumor settings. In EMT-6 breast cancer model, combination therapy enhanced antitumor efficacy relative to either monotherapy, induced the generation of tumor-specific immune memory, and enhanced T-bet expression and proliferation of NK and CD8+ T-cells (65). Furthermore, a phase 1b/2 study (NCT02554812), which incorporates a group of TNBC patients, is currently testing Avelumab combination with novel immunotherapies.

Improving the treatment of TNBC remains still a considerable challenge. Our *in vitro* results suggest that Avelumab-mediated ADCC could be a useful mechanism to eliminate tumor cells in TNBC patients, apart from the blockade of the PD-1/PD-L1 pathway. By this approach, Avelumab could act as a tumor antigen-targeting antibody and checkpoint inhibitor at the same time, resulting in NK and CD8 T cell activation. Avelumab combination with immunomodulators such as IL-15 or IL-2 could be taken into consideration to increase the therapeutic efficacy of Avelumab in TNBC.

## Ethics statement

This study was carried out in accordance with the recommendations of Comité de Ética del Instituto Medico Especializado Alexander Fleming. Blood samples from healthy donors were obtained after signing informed consent, according to the Instituto Medico Alexander Fleming guidelines, at the Hemotherapy Service.

## Author contributions

EJ collected, assembled, analyzed and interpreted the data, and wrote the manuscript. AA and MP collected and analyzed the data. JM interpreted the data and revised the manuscript. EL analyzed and interpreted the data and wrote the manuscript. All authors contributed to manuscript revision, read, and approved the submitted version.

### Conflict of interest statement

This study was supported by Merck KGaA, Darmstadt, Germany as a grant for an investigator-sponsored study between Merck KGaA and Fundación Cáncer. Merck KGaA reviewed the manuscript before journal submission. The authors are fully responsible for the content of this manuscript, and the views and opinions described in the publication reflect solely those of the authors.

## Abbreviations

PD-L1: Programmed death-ligand 1
TNBC: Triple Negative Breast Cancer
PD-1: Programmed cell death protein 1
mAb: Monoclonal Antibody
ADCC: Antibody-directed cellular cytotoxicity
IFN-γ: Interferon-gamma
IL-2: Interleukin 2
IL-15: Interleukin 15
NK: Natural Killer
TIL: Tumor-infiltrating lymphocytes
BC: Breast cancer
PTEN: Phosphatase and tensin homolog
PI3K: Phosphoinositide 3-kinase
Ig: Immunoglobulin
Fc: Fragment crystallizable
FcγR: Fc gamma Receptor
TNF-α: Tumor necrosis factor alpha
HLA: Human Leucocyte Antigen
MHC: major histocompatibility complex
FCS: fetal calf serum
MICA/B: MHC class I related-protein A and B
MFI: Median fluorescence intensity
PBMC: Peripheral blood mononuclear cells
ON: overnight
NK:BC ratio: NK cell: BC cell ratio
ADCR: Antibody-dependent cytokine release
γc: γ-chain
Treg: Regulatory T cells
STAT5: signal transducer and activator of transcription 5
BCL2: B-cell lymphoma 2.
