# Supplementary material for: Avelumab, an IgG1 anti-PD-L1 Immune Checkpoint Inhibitor, Triggers NK Cell-Mediated Cytotoxicity and Cytokine Production Against Triple Negative Breast Cancer Cells
Source: Front Immunol. 2018 Sep 20;9:2140. doi: 10.3389/fimmu.2018.02140 (PMC6159755; doi:10.3389/fimmu.2018.02140)
Supplement: Supplementary file 1 [file Data_Sheet_1.PDF]

Supplementary Material

Avelumab, an IgG1 anti-PD-L1 immune checkpoint inhibitor, triggers NK cell-mediated cytotoxicity and cytokine production against Triple Negative Breast Cancer cells

Estefanía Paula Juliá[1], Analía Amante[1], María Betina Pampena[1], José Mordoh[1,2,3] and Estrella Mariel Levy[1]*.

Supplementary Figure 1. IFN-γ effect on Avelumab-triggered degranulation against BT-549 cells. NK cell degranulation against IFN-γ treated or untreated BT-549 cells coated with IgG1 or Avelumab. Basal degranulation without target cells is shown in light blue symbols. Each symbol represents an individual donor. Bars with different letters are statistically different, p<0.05 (n=6, ANOVA).

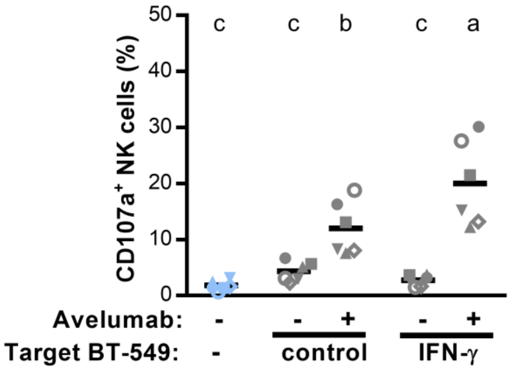

Supplementary Figure 1

Supplementary Figure 2. NK cell degranulation and cytokine production by CD56^{dim} and CD56^{bright} NK cell subsets (A) Representative gating strategy: Lymphocytes were gated using FSC-A vs SSC-A plot, single cells using FSC-A vs FSC-H plot, and then NK cells were defined as CD3^{-}CD56^{+} cells. CD56^{dim} and CD56^{bright} NK cell subsets were gated according to the expression of CD56. **(B)** PBMC were stimulated ON with 1000 U/ml IL-2 or 10 ng/ml IL-15, or left untreated, and then cultured without target cells or with MDA-MB-231 coated with IgG1 or Avelumab. Percentage of CD56^{dim} and CD56^{bright} NK expressing IFN-γ (upper panel), TNF-α (middle panel) and CD107a (lower panel) are shown. Bars with different letter are statistically different, p<0.05 (n=6, ANOVA). **(C)** Representative dot plots showing the percentage of CD56^{dim} (in blue numbers) and CD56^{bright} (in red numbers) NK cells expressing IFN-γ, TNF-α and CD107a against Avelumab-coated MDA-MB-231 cells.

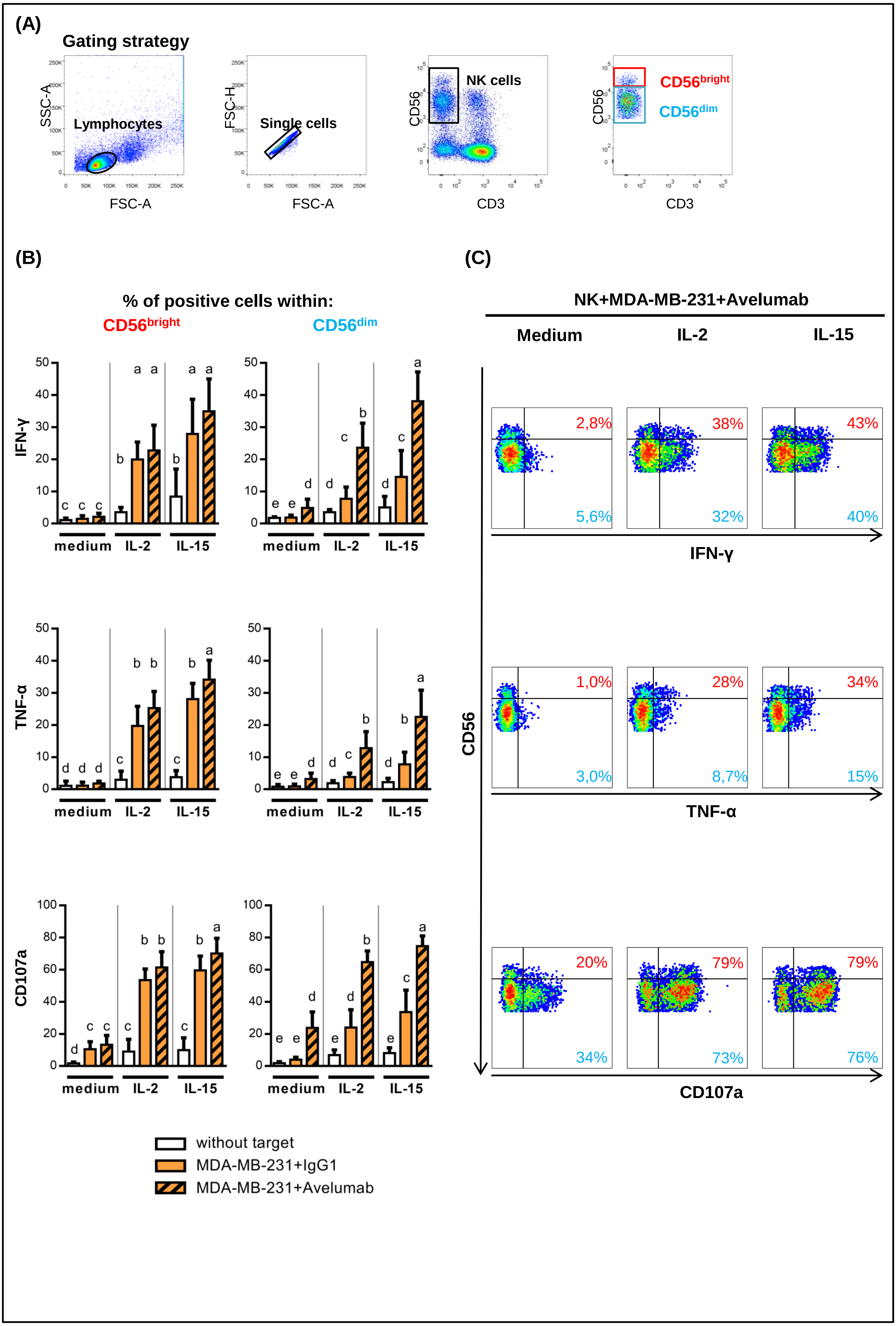

Supplementary Figure 2
